# Outcomes of non-small cell lung cancer patients with non-V600E *BRAF* mutations: a series of case reports and literature review

**DOI:** 10.3389/fonc.2024.1307882

**Published:** 2024-03-27

**Authors:** Raluca Lazar, Cathie Fischbach, Roland Schott, Laura Somme

**Affiliations:** Oncology Department, Institut De Cancérologie Strasbourg-Europe, Strasbourg, France

**Keywords:** NSCLC, *BRAF* non-V600E mutations, immunotherapy, targeted therapy, PD-L1

## Abstract

Non-small cell lung cancer (NSCLC) is the most prevalent form of lung cancer, accounting for approximately 85% of cases of lung cancer. The standard first-line therapy for patients without oncogenic driver metastatic NSCLC is anti PD-L1 immune checkpoint inhibition (ICI) with platinum-based chemotherapy. Approximately 4% of NSCLC patients harbor *BRAF* mutations; the V600E mutation is the most common. Non-V600 mutations is an heterogeneous population and account for approximately 50% of *BRAF*-mutated NSCLC. *BRAF* mutations are classified into 3 functional classes based on their kinase activity and their signaling mechanism. The European Medicines Agency and the United States Food and Drug Administration have approved dabrafenib, an anti-BRAF tyrosine kinase inhibitor (TKI), in combination with trametinib, an anti-MEK TKI, for the treatment of patients with *BRAF* V600E-mutated metastatic NSCLC. The use of targeted therapies in NSCLC with *BRAF* non-V600E mutations remains controversial. There is a lack of guidelines regarding therapeutic options in non-V600E *BRAF*-mutated NSCLC. Herein, we presented 3 cases of NSCLC with *BRAF* non-V600E mutations and reviewed the current state of therapies for this particular population of lung cancer.

## Introduction

Lung cancer is a major contributor to global cancer-related deaths in 2023 (1). Non-small cell lung cancer (NSCLC) accounts for 85% of all lung cancers (2). The standard of care in NSCLC without oncogenic driver alterations is platinum-based chemotherapy combined with the immune checkpoint inhibitor anti-programmed death protein/ligand 1 (PDL1); this treatment leads to a median overall survival of 15.9 months in the metastatic stage. The advent of precision medicine, whereby the identification of oncogenic driver alterations, such *EGFR* alterations or *ALK* rearrangements, confers sensitivity to tyrosine kinase inhibitors (TKI), has revolutionized the prognosis of NSCLC in this particular population (3). V-Raf murine sarcoma viral oncogene homologue B1 (*BRAF*), one of the three members of the RAF kinase family (along with ARAF and CRAF), belongs to the group of serine/threonine kinases that lies downstream of RAS and directly activates MEK 1/2, resulting in ERK1/2 phosphorylation (4). Activating mutations in the *BRAF* gene represent an oncogenic driver that leads to constitutive kinase activity, which triggers downstream pathways regulating cancer cell growth, survival, proliferation and differentiation (5). *BRAF* mutations are typically grouped into 3 functional classes based on both signaling mechanism and kinase activity. Class 1 mutations signal RAS-independent active monomers (e.g., V600E) with constitutively strong kinase activity; class 2 mutations signal constitutively active RAS-independent dimers with either high or intermediate kinase activity (e.g., K601E, L597V/Q/R, G469V/S/R/E/A, G464V); and class 3 mutations signal RAS-dependent heterodimers with low or absent kinase activity (e.g., G596R, D594Y/N/G/E, N581Y/S/I, G466V/L/E/A, D287Y) (6).

The EMA and the United States FDA have approved the use of dabrafenib, an anti-BRAF TKI, in combination with trametinib, an anti-MEK TKI, to treat patients with *BRAF* V600E-mutated metastatic NSCLC (7). However, the use of targeted therapies in NSCLC with *BRAF* non-V600E mutations remains controversial. Literature data, evaluating clinical characteristics and therapeutic management in a *BRAF* non-V600E mutated NSCLC population, is poor.

This article reports three cases of NSCLC with *BRAF* non-V600E mutation and reviews the current state of art for this particular population of lung cancer, which lacks a standard of care due to limited clinical data.

## Case presentation

**Figure 1:**
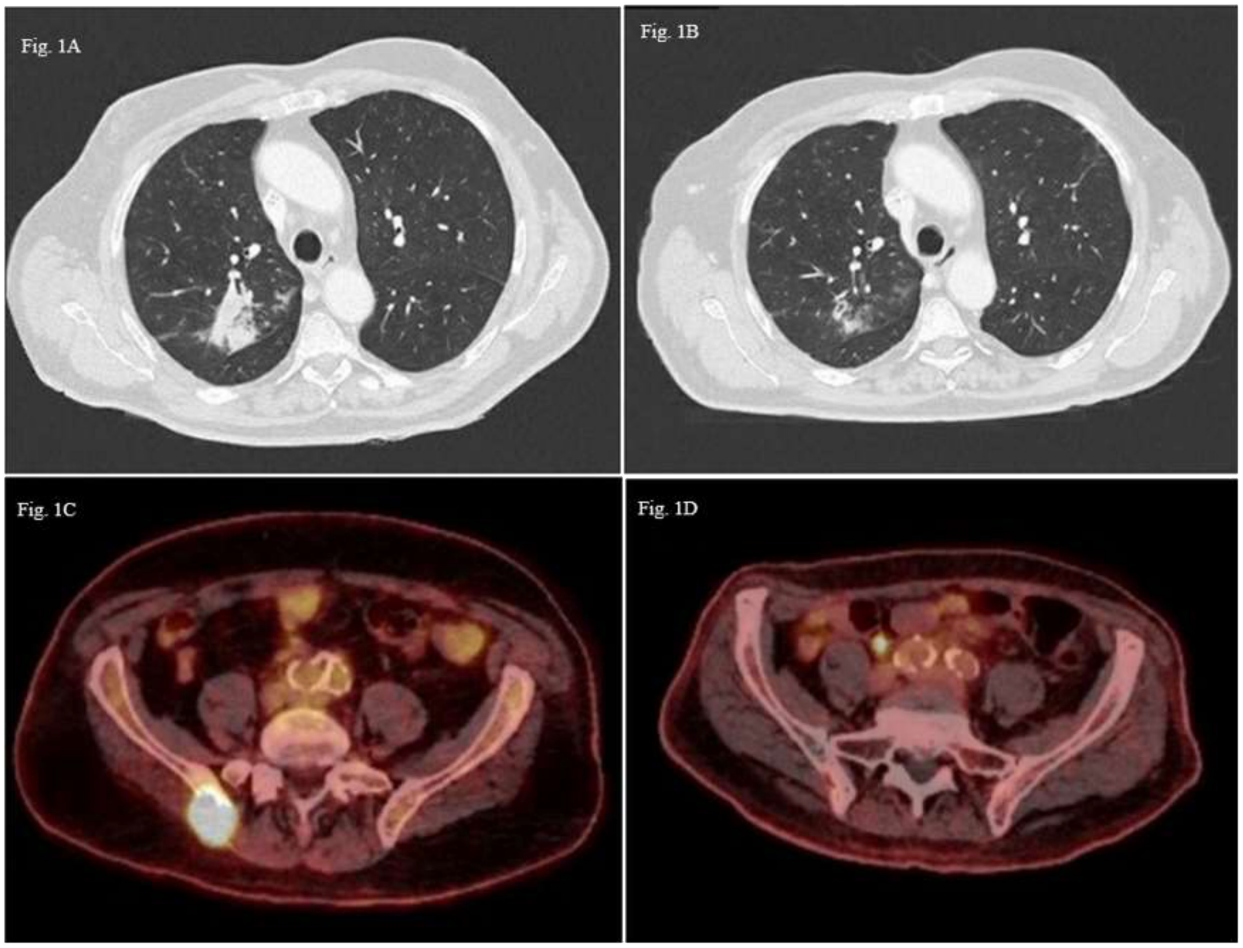
Thoracic CT-scan images axial sections **(A)** Pulmonary lesion in the posterioir right upper lobe (48mm); **(B)** Partila regression of the pulmonary lesion (22 mm); 18F-FDG PET scan images axial sections **(C)** Hypermetabolic bone lesion of the right iliac spine; **(D)** Partial metabolic response of the bone lesion.

Patient A: A 76-year-old male patient, a former smoker with a medical history of colon cancer treated by surgery and adjuvant capecitabine in 2013, was diagnosed with right upper lobe lung adenocarcinoma classified cT3N1M0 (8^th^ TNM edition). Histopathological analysis revealed poorly differentiated *ALK -, EGFR - PDL1* positive (100% in IHC) adenocarcinoma without oncogenic drivers. Cerebral CT scan was performed without cerebral metastasis. Chemotherapy based on carboplatin (AUC5) and pemetrexed (500 mg/m2) in combination with pembrolizumab (200mg) was introduced as neoadjuvant therapy with a significant regression after four cycles of treatment. Right upper lobectomy was performed and confirmed residual acinar-type infiltrated adenocarcinoma with significant necrotic and chronic fibro-inflammatory classified ypT1aN0. Nine months later, the patient experienced seizures. Due to neurological symptoms, a brain magnetic resonance imaging (MRI) was performed and revealed appearance of two cerebral metastases (right cerebellum hemisphere and right frontal region). The patient presented concomitant extra-cerebral relapse with a nodule in the posterior segment of the right upper lobe (48 mm) (**Figure 1A**) and an osteolytic lesion of the right sacral. Cerebral stereotactic brain radiotherapy of both metastases (33 Gy in three fractions of 11 Gy) and chemo-immunotherapy (platinum-based chemotherapy and pembrolizumab) was rechallenged as first line metastatic therapy. After two cycles, the revaluation showed regression of the right upper nodule to 22 mm *versus* the initial 48 mm (**Figure 1B**), but due to digestive (grade 2 anorexia, grade 2 mucositis) and hematological toxicity (grade 4 thrombocytopenia), chemotherapy was stopped, and pembrolizumab monotherapy was continued. Six cycles later, ^18^F-FDG PET scan showed metabolic mediastinal, supraclavicular and hilar lymph nodes and bone progression in the right iliac spine (**Figure 1C**). Due to chemo-immunotherapy resistance a next-generation sequencing of circulating tumoral cDNA was performed and revealed the *BRAF G596R* in exon 15. Paclitaxel (80 mg/m^2^) and Bevacizumab (10 mg/kg) as second-line treatment allowed a partial bone response and a complete lymph node response during eleven months (**Figure 1D**). Chemotherapy was stopped due to side effects (nephrotic syndrome and grade 3 neuropathy). After six months of observation, tumoral disease progressed and metronomic navelbine was proposed, as the patient requested oral treatment. The treatment was stopped after 1 month due to adverse effects. After two administrations of paclitaxel (90mg/m2), it was stopped due to poor tolerance. The patient is still alive four years after diagnostic, in the last 3 months with best supportive care.

**Figure 2:**
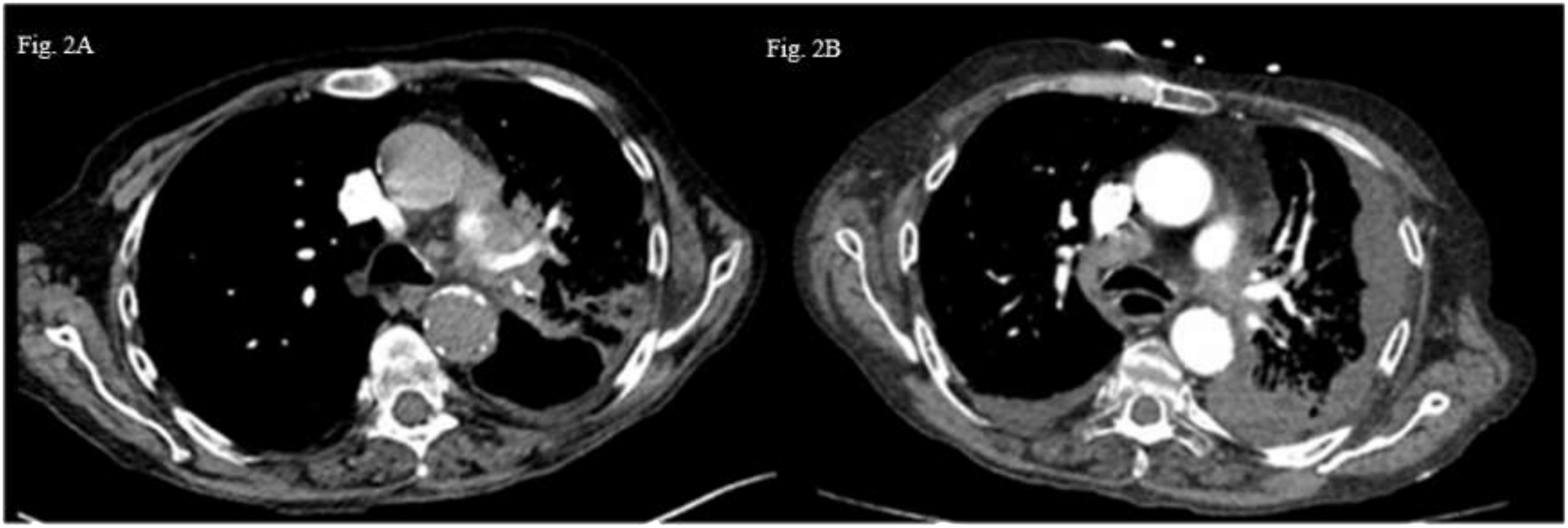
Thoraric CT-scan images axial section **(A)** Left hilar tumoral lesion. **(B)** Appearance of a left plural effusion and progression lower right paratracheal area lymph nodw (4R).

Patient B: A 60-year-old male patient with a medical history of supraglottic laryngectomy due to squamous cell carcinoma, chronic obstructive lung disease, ongoing smoking and chronic alcoholism was diagnosed with pulmonary and brain metastatic lung adenocarcinoma (cT4N3M1c according to 8^th^ TNM edition) (**Figure 2A**). Histological analysis revealed poorly differentiated PD-L1 negative adenocarcinoma and next-generation sequencing of circulating tumoral cDNA revealed *BRAF G464V* mutation without other oncogenic alterations. Weekly carboplatin (AUC2) and paclitaxel (80 mg/m^2^) chemotherapy due to respiratory and general fragility was proposed during two cycles without efficacy (**Figure 2B**). The patient received palliative care, and he passed away in the subsequent weeks.

**Figure 3:**
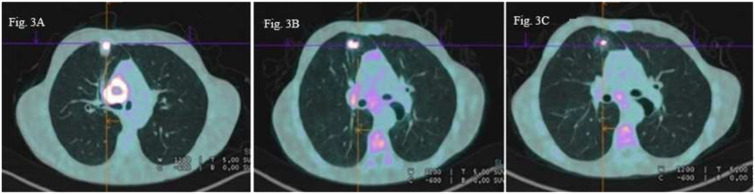
18F-FDG PET scan images axial sections **(A)** Right upper lobe pulmonary primary lesion (26×19 mm) with right hilar necrotic adenophaty **(B)** Partial metabolic regression of the primary lung lesion (10 mm versus 26 mm and the right hilar lymph node **(C)** Persistent partial metabolic response of the pulmonary lesion and hilar lymph node.

Patient C: In December 2022, a 50-year-old male patient with a medical history of polyaddiction (tobacco, cannabis, and heroin) and hepatitis C presented with left hemiparesis and headaches. Brain MRI revealed a right frontal brain metastasis treated by surgery. An ^18^F-FDG PET scan revealed a right pulmonary upper lobe primary lesion measuring 26 x 19 mm associated with right hilar necrotic adenopathy (cT3N1M1c according to 8^th^ TNM edition) (**Figure 3A**). Histological analyses of brain lesion revealed cerebral metastases of a PD-L1 negative lung adenocarcinoma and next-generation sequencing of circulating tumoral cDNA revealed *BRAF* G469V mutation. Cerebral stereotactic radiotherapy (33 Gy in three fractions of 11 Gy) followed by chemotherapy based on carboplatin (AUC5) and pemetrexed (500 mg/m^2^) was started. A checkpoint inhibitor was not administered due to a history of hepatitis C. After two cycles of systemic treatment, partial response was observed in the primary lung lesion (10 mm *versus* 26 mm), lymph nodes and right frontal lesions (**Figure 3B**). Partial response was confirmed after four additional cycles (**Figure 3C**). The patient is still receiving maintenance treatment with pemetrexed monotherapy.

## Discussion

*BRAF* mutations define a specific molecular type of NSCLC, accounting 3-5% of cases. *BRAF* is a serine/threonine protein kinase that belongs to the RAF kinase family, along with other isoforms ARAF and CRAF, upon activation by RAS, these proteins play a crucial role in growth, proliferation, migration, and survival by activating the MAPK-ERK pathway. Oncogenic mutations in components of this pathway lead to the constant activation of the MAPK-ERK cascade and oncogenic transformation (8). *BRAF* mutations are classified intro three classes according to their dimerization status, their kinase activity, and RAS dependence for activation; class 1 contains mutation of codon 600 on exon 15, and are characterized by RAS-independent, high BRAF kinase activity in a monomeric status. Class 2 are located in exon 11 and 15 and result in RAS-independent homodimers with high/intermediate kinase activity and class 3 mutants have absent or low kinase activity (8).

There are few data regarding the clinical and survival outcomes of NSCLC patients with non-V600E *BRAF* mutations. *BRAF*-mutated NSCLC patients are commonly males and smokers (9). Tissot et al. examined 38 patients with NSCLC non-V600E *BRAF* mutations, and a high proportion of them were smokers (92%) (10). In contrast, patients with *BRAF* class 1 mutations NSCLC were more likely to be never smokers (11). In accordance with the literature, the three included patients with NSCLC with *BRAF* class 2 and 3 mutations were active smokers.

A retrospective analysis of 107, 75 and 54 patients with class 1, 2 and 3 *BRAF*-mutated lung cancer, respectively, revealed that patients with class 2 or 3 mutations were more likely to have brain metastases than those with class 1 mutations (29% and 31% for class 2 and 3 *versus* 9% class 1) (11). In accordance with the literature, two of the three reported cases developed brain metastasis. In a retrospective analysis of 236 patients diagnosed with NSCLC *BRAF-*mutated, brain metastases were detected at diagnosis in 29% (n=69). *BRAF* class 2 and class 3 mutations were associated with high risk of cerebral metastases at diagnosis compared with class 1 alterations (p=0.011 class 1 *versus* class 2; p=0.007 class 1 *versus* class 3) (11). The patient C harboring *BRAF* class 3 mutation (*BRAF G469V*) had brain metastases at the diagnosis and patient A harboring also a *BRAF* class 3 mutations (*BRAF* mutation *G596R*) developed cerebral metastasis after 9 months of follow-up.

Considering survival outcomes in terms of the type of *BRAF* mutation, a retrospective study including 80 *BRAF* mutated-NSCLC consisting of V600E in 42 (53%) cases; non-V600E in 38 (48%) cases reported a longer overall survival (OS) in patients with *BRAF* V600E mutations than in those with non-V600E mutations (25 *versus* 13 months, p=0.153) (10). Overall survival did not significantly differ regarding *BRAF* class 2 or 3 mutations (10). The median OS was 3 years, 3 months and 7 months in patients A, B and C, respectively.

Considering therapeutic data, chemotherapy has shown limited results in patients with previously untreated (class 1, 2 and) *BRAF*-mutated NSCLC. In a retrospective analysis, survival outcomes for patients treated with carboplatin and pemetrexed who had a class 2 and 3 BRAF-mutated NSCLC were unfavorable compared to class 1 *BRAF* mutation; median PFS and OS were 4.9 months and 15.6 months for class 3, 3.3 months and 13.9 months for class 2 and 6.2 months and 40.1 months class 1 (11). Chemotherapy with carboplatin and pemetrexed was effective in patient C with *BRAF* G469V mutation (7-month PFS).

Anti-*BRAF* and anti-*MEK* were approved in V600E *BRAF*-mutated NSCLC, but there are few data about the use of these TKIs among the non-V600E *BRAF* population (**Table 1**) (19). The use of targeted therapies in non-V600E *BRAF*-mutated NSCLC remains controversial (6). In the AcSe trial NSCLC cohort, the disappointing of *BRAF* inhibition alone against non-V600 *BRAF* mutation was confirmed among 17 patients who received treatment with vemurafenib, no response was observed with a median PFS of 1.8 months (12). In the EURAF cohort amongst the six patients with *BRAF* non-V600 mutation, only one with *BRAF* G569V mutation responded to vemurafenib or dabrafenib (anti-BRAF TKI) (13). Gautschi et al. reported a case of patient with *BRAF G469L* mutated advanced NSCLC treated with vemurafenib with no response (20). In contrary, Dagogo et al. presented a case of a patient with a *BRAF G469A* mutated NSCLC with a durable response to the combination dabrafenib with trametinib (14). Su et al. presented a durable response to combination of dabrafenib and trametinib in a patient with *BRAF K601E* mutated lung adenocarcinoma (15). Reyes et al. reported a sensitivity to BRAF/MEK inhibitors in a *G469A* and *W604C* non-V600E *BRAF* mutant lung adenocarcinoma, resulting in a benefit of 15 months (16). In our case the patient A who required the chemotherapy to be interrupted due to side effects, at progression one of the therapeutic options could have been BRAF/MEK inhibition but it was not considered as it is not approve in Europe.

**Table 1:** Target therapies outcomes in advanced *BRAF* non-V600 NSCLC.

Study	Type	Drug	Patients (n)	Mutations	Response	Median PFS months (95% Cl)
AcSe (NSCLC cohort) (12)	Phase II	Vemurafenib (>= first line)	17	3 K601E 3 G469A 3 G466V 3 N581S 2 K601N 1 G469V 1 G466A 1 G569R	No response	1.8 (1.4-2.1)
EURAF cohort (13)	Retrospective	Vemurafenib, Dabrafenib (median third line)	6	1 G466V 1 G469A 1 G469L 1 G596V 1 V600K 1 K601E	1 PR (G596V)	NA
Dagogo-Jack et al. (14)	Case report	Dabrafenib + trametinib (fourth line)	1	G469A	PR (DoT 6 m)	NR
Su et al. (15)	Case report	Dabrafenib + Trametinib (first line)	1	K601E	PR (DoT 9 m)	NR
Reyes et al. (16)	Case report	Dabrafenib + Trametinib (first line)	1	G469A	PR	NR
Sereno et al. (17)	Case report	Sorafenib (>fourth line)	1	G469R	PR (DoT 6 m)	NA
Sen et al. (18)	Case report	Dasatinib (first line)	1	Y472C	CR (DoT 12 w, DoR 4y)	NR

CR, complete response; DCR, disease control rate; DoT, duration of treatment; DoR, duration of response; NA, not available; NR, not reached; NSCLC, non-small cell lung cancer; PFS, progression-free survival; ORR, overall response rate; PR, partial response; pts, patients.

In the BELIEVE trial an objective response rate of 28% and a disease control rate of 84% in 50 patients with solid tumors harboring V600E/R or non-V600E *BRAF* mutations treated with dabrafenib and trametinib, were reported (21).

Beside the activity of BRAF/MEK inhibition, two cases showed efficacy of sorafenib, an oral multiple tyrosine kinase inhibitor, against *BRAF G469V* and *G469R* mutations (22). Moreover, a complete response lasting over 4 years was reported in a patient with *BRAF* Y472C mutated NSCLC treated with dasatinib, a BCR-ABL tyrosine kinase inhibitor (18).

Further research is needed to better understand the specific molecular pathways that are compromised in non-V600 mutations to develop specific targeted therapies.

Immune-checkpoint inhibitors have changed the landscape of lung cancer treatment over the past decade but proving a clear overview on the efficacy of ICI in *BRAF* mutant NSCLC is challenging given the absence of prospective data, the small population included in retrospective/observational studies, and their fragmentation based on different mutation classes (23). In a retrospective multi-centric study conducted by Dudnik et al., the association between *BRAF* mutation and PD-L1 expression was investigated in 39 NSCLC patients (21 patients with BRAF V600E mutation and 18 patients with BRAF non-V600E mutation). The *BRAF* mutation was associated with a higher level of PD-L1 expression (between 42% and 50%) than the 28% prevalence in the overall population of NSCLC patients (24). Differences in the distribution of PD-L1 between the *BRAF* V600E and non-V600E groups were observed*, BRAF* V600E expressed more PD-L1 (24). In contrast, Negrao et al. showed that the rate of PD-L1 positivity (TPS ≥1%) in the V600E genotype (75.4%) was higher than that in non-V600E genotypes (55.8%) (25). Regarding the results of immunotherapy, the median PFS and OS of the non-V600E *BRAF* population were 5.4 months and 14.9 months, respectively (25). A retrospective study including 18 patients with *BRAF* non-V600E, the median PFS was 4.1 months (5). A retrospective, multi-centric study showed that 18 patients harboring *BRAF* non-V600E mutation achieved 4.9-month median PFS and 12-month median OS (26). In the Immunotarget study comparable outcomes (27). In a population of 98 patients with non-V600E mutated NSCLC patients, ORR to immunotherapy was 22% with a median OS of 17.2 months (28). A population of 37 patients with non V600E *BRAF*-mutant NSCLC, reported by Murciano-Goroff et al, treated with ICI, showed an ORR of 26% (29). A case of NSCLC harboring a rare BRAF E501Q mutation and PD-L1 negative expression reported a durable response after first-line treatment with ICIs and pemetrexed; the patient was alive 38 months after treatment initiation (30). Rittberg et al. demonstrated prolonged disease control over 4 years with ICI monotherapy in a 61-year-old patient with advanced NSCLC harboring the *BRAF G469A* mutation and PD-L1 expression >50% (31). In patient A, who had NSCLC with the *BRAF* mutation *G596R* exon 15, PFS was 4.5 months.

Immune-based therapy (**Table 2**) could be an appropriate option for treating lung adenocarcinoma with a non-V600E *BRAF* mutation. Furthermore, prospective clinical studies are necessary to determine the effectiveness of using immune-based therapy based on the *BRAF* mutation class.

**Table 2:** ICIs in *BRAF*-mutated NSCLC.

Study	Patients (n)	Treated with ICIs (n)	Line of treatment	Drugs	Median OS, months (95% Cl)	Median PFS, months (95% Cl)	ORR
Dudnik et al. (5)	39 21 V600E 18 non-V600E	22 12 V600E 10 non- V600E	First to third	Pembrolizumab Nivolumab Atezolizumab	V600E NR non-V600E NR p=0.53	V600E 3.7 (1.6–6.6) non-V600E 4.1 (0.1-19.6) p=0.37	V600E 3.7 (1.6–6.6) non-V600E 4.1 (0.1–19.6) p=0.37
Mazieres et al. (27)	43 17 V600E 18 non- V600E 8 unknowns	43	First and further	anti-PD-(L)1	V600E 8.2 (11–NR) non-V600E 17.2 (2.7–NR) *p*=0.28	V600E 1.8 (1.0–4.6) non-V600E 4.1 (2.9–9.0) *p*=0.20	V600E and others 24% (9/37 evaluable)
Guisier et al. (32)	44 26 V600 18 non-V600	44	First and further (42 pre-treated patients)	anti-PD-(L)1	V600 22.5 (8.3–NR) non-V600 12.0 (6.8–NR)	V600 5.3 (2.1–NR) non-V600 4.9 (2.3–NR)	V600 26% non-V600 35%
Offin et al. (28)	177 41 V600 136 non-V600	46 36 V600 10 non-V600	Second (median)	Pembrolizumab Nivolumab Atezolizumab Nivolumab + ipilimumab	Non-V600 2.4	Not reported	V600 10% non-V600 22% *p*=0.66
Murciano- Goroff et al. (29)	127 29 class 1 36 class 2 23 class 3 39 VUS	50 13 class 1 37 class 2/3	First and further	Pembrolizumab Nivolumab Atezolizumab Nivolumab + ipilimumab Experimental	NA	NA	Class 1 9% class 2/3 26% *p*=0.25

DoR, duration of response; (m)OS, (median) overall survival; (m)PFS, (median) progression-free survival; NA, not available; NR not reached; ORR, objective response rate; PD-(L)1, programmed cell death-(ligand)1.

## Conclusion

Patients with non-V600 *BRAF* mutations in NSCLC constitute a seldom-encountered yet clinically distinct subgroup. The challenge lies in the insufficiently treatment options attributable to the low prevalence of this population. Employing molecular profiling techniques such as next-generation sequencing, genomics and single-cell sequencing may prove instrumental in identifying mutations and resistance pathways for improved patient outcomes. *BRAF* non-V600 mutations include a diverse category with varied responses to targeted agents, as indicated by existing evidence. Lacking prospective evidence, promoting the publication of case reports or series about clinical experience with targeted agents for specific molecular alterations and corresponding patient outcomes is advisable. Conversely, individuals with *BRAF* mutation may experience improved outcomes when treated with immunotherapy, prospective evidence regarding the outcomes associated with immunotherapy+/- chemotherapy in *BRAF* mutant disease is awaited; this will help guide first-line treatment decisions.

The treatment of NSCLC patients harboring the non-V600E *BRAF* mutation is still an area of active research, and multidisciplinary teams of oncologists and researchers are working to develop novel therapies that can improve outcomes for this population.

## Data availability statement

The raw data supporting the conclusions of this article will be made available by the authors, without undue reservation.

## Ethics statement

Written informed consent was obtained from the individual(s) for the publication of any potentially identifiable images or data included in this article.

## Author contributions

RL: Writing – original draft. RS: Writing – review & editing, Visualization, Resources. CF: Writing – review & editing, Visualization, Resources. LS: Writing – review & editing, Visualization, Validation, Supervision, Resources, Project administration, Funding acquisition, Conceptualization.
